# Systematic Literature Review on the Impact of COVID-19 Pandemic on Referrals to Child and Mental Health Services (CAMHS) in United Kingdom (UK)

**DOI:** 10.1192/bjo.2024.411

**Published:** 2024-08-01

**Authors:** Chetana Patil

**Affiliations:** NHS Orkney, Kirkwall, United Kingdom. NHS Grampian, Aberdeen, United Kingdom

## Abstract

**Aims:**

Child and Adolescent Mental Health Services (CAMHS) is a highly specialised service to which children with severe mental health problems are referred. The COVID-19 pandemic brought with it a lot of uncertainty, and healthcare systems across the UK struggled to cope with the added pressure. The aim of this systematic review is to analyse the literature exploring the effects of the COVID-19 pandemic on the severity of mental health conditions and referral rates to CAMHS services in the UK. The findings from this study will help the services understand the impact of the pandemic on referral rates to CAMHS, the severity of various mental health conditions, and how the services are managing.

**Methods:**

An extensive search, following PRISMA guidelines, was undertaken across multiple electronic databases using a predetermined search strategy. Studies reporting on mental health conditions in children post-pandemic and on referral rates to CAMHS in the UK were included. Subsequently, data extraction, quality appraisal and qualitative analysis were performed in a descriptive style.

**Results:**

Initially, referrals to CAMHS decreased during the first lockdown, followed by a significant increase in referrals throughout the pandemic period. The referral rate to CAMHS remains steady until adolescence, with a rapid increase in referrals to the services during the teenage years. More adolescent girls were referred to CAMHS compared with boys and are at an increased risk of developing mental health conditions. A higher number of children and young persons presented with urgent referrals and heightened symptoms during the pandemic compared with the pre-pandemic levels. In particular, there was a significant increase in children presenting with eating disorder problems, accompanied by an increased severity of symptoms. Furthermore, there was an observed rise in depression and anxiety among children and young people, along with an increase in the use of antidepressant medication.

**Conclusion:**

Referrals to CAMHS increased during the pandemic, with increased severity of symptoms observed, particularly in children and young people with eating disorders and neurodevelopmental conditions. Future research should explore the enduring impact of the pandemic on referral rates and presentations to CAMHS. This exploration is essential to aid senior managers and policymakers in decision-making, enabling the implementation of appropriate measures to manage the pattern of demands on CAMHS and shape the future service delivery of CAMHS in the UK.

